# Urine Monocyte Chemoattractant Protein-1 and Lupus Nephritis Disease Activity: Preliminary Report of a Prospective Longitudinal Study

**DOI:** 10.1155/2015/962046

**Published:** 2015-07-12

**Authors:** Sabah Alharazy, Norella C. T. Kong, Marlyn Mohd, Shamsul A. Shah, Arbaiyah Ba'in, Abdul Halim Abdul Gafor

**Affiliations:** ^1^Nephrology Unit, Department of Medicine, Universiti Kebangsaan Malaysia Medical Centre, 56000 Kuala Lumpur, Malaysia; ^2^Department of Medical Microbiology & Immunology, Universiti Kebangsaan Malaysia Medical Centre, 56000 Kuala Lumpur, Malaysia; ^3^Department of Community Medicine, Universiti Kebangsaan Malaysia Medical Centre, 56000 Kuala Lumpur, Malaysia

## Abstract

*Objective*. This longitudinal study aimed to determine the urine monocyte chemoattractant protein-1 (uMCP-1) levels in patients with biopsy-proven lupus nephritis (LN) at various stages of renal disease activity and to compare them to current standard markers. *Methods*. Patients with LN—active or inactive—had their uMCP-1 levels and standard disease activity markers measured at baseline and 2 and 4 months. Urinary parameters, renal function test, serological markers, and renal SLE disease activity index-2K (renal SLEDAI-2K) were analyzed to determine their associations with uMCP-1. *Results*. A hundred patients completed the study. At each visit, uMCP-1 levels (pg/mg creatinine) were significantly higher in the active group especially with relapses and were significantly associated with proteinuria and renal SLEDAI-2K. Receiver operating characteristic (ROC) curves showed that uMCP-1 was a potential biomarker for LN. Whereas multiple logistic regression analysis showed that only proteinuria and serum albumin and not uMCP-1 were independent predictors of LN activity. *Conclusion*. uMCP-1 was increased in active LN. Although uMCP-1 was not an independent predictor for LN activity, it could serve as an adjunctive marker when the clinical diagnosis of LN especially early relapse remains uncertain. Larger and longer studies are indicated.

## 1. Introduction

Lupus nephritis (LN) contributes to significant morbidity and mortality in patients with systemic lupus erythematosus (SLE) [1, 2]. Renal biopsy is the gold standard for diagnosis of LN. However, repeated biopsies are not always practical in real life practice especially in patients with frequent relapses or in those with associated severe haematologic or cerebral manifestations. Moreover, renal biopsy is a relatively invasive procedure and is associated with a significant albeit small risk, particularly in those patients who may have undiagnosed coagulopathy, for example, presence of antiphospholipid antibodies/antiphospholipid syndrome, or are on anticoagulants [3].

Active LN especially early flares/relapses often respond to appropriate treatment with immunosuppressive agents. However, these drugs are themselves associated with significant morbidity and even mortality whilst uncontrolled LN activity leads to chronic or end stage kidney disease (ESRD) and even death. Current standard laboratory markers such as proteinuria cannot always distinguish between active and inactive renal disease especially in patients with a recent history of LN [4]. These tests also lack sensitivity and specificity for the monitoring of LN activity especially early flares. Hence, it is essential to identify noninvasive new biomarkers that are able to predict renal flares/relapses as well as reflect the severity of LN activity. These biomarkers could be followed serially and may enable timely institution of appropriate treatment before the development of significant inflammatory injury in the kidney.

Monocyte chemoattractant protein-1 (MCP1) is a chemokine that attracts monocytes/macrophages to sites of inflammation [5]. MCP-1 is produced by mesangial, podocyte, and monocyte cells in response to various proinflammatory stimuli such as tumor necrosis factor alpha (TNF-*α*). These inflammatory cells and substances subsequently mediate tissue injury and contribute to the development of renal dysfunction. Moreover, MCP-1 binding has been shown to reduce levels of nephrin, an important protector of kidney cell function [6] whereas antagonists to MCP-1 prevent renal disease progression in murine models. Marks et al. [7] showed that the presence of MCP-1 within the glomerulus correlated with a poor renal prognosis and could identify more severe histological classes of LN in paediatric patients.

Several studies have shown that the urine levels of MCP-1 were significantly greater in patients undergoing a renal flare than in patients with stable renal disease or healthy controls [8–10].

We have previously reported that, in a cross-sectional study of 100 adult SLE patients with LN, uMCP-1 levels did reflect LN activity [11]. In this paper, we present the preliminary results of our prospective follow-up study which evaluated uMCP-1 as a potential marker for LN response to treatment and/or early relapse in this same LN patient cohort.

## 2. Methods

The same 100 LN patients whose baseline data had been previously reported by us [11, 12] were followed in a prospective longitudinal fashion at 2 and 4 months. All patients fulfilled the ACR classification criteria for SLE [13] and eligibility included all those with biopsy-proven LN regardless of activity status at recruitment. We excluded LN patients with ESRD or who required chronic dialysis or had undergone renal transplantation and those with clinical LN in whom a renal biopsy could not be performed as well as pregnant patients. The patients were divided into two groups based on the presence or absence of LN activity as detailed below. The active LN group included those with active renal disease or nonremission (NR) or who had a relapse/flare. The inactive LN group included those in complete or partial remission (CR/PR). The calculated sample size was 100 patients [11]. Informed consent was obtained from all recruited subjects. The study protocol was approved by the Medical Research and Ethics Committee of the Universiti Kebangsaan Malaysia Medical Centre (UKMMC).

### 2.1. Definition of LN Activity

- Active LN was defined by the presence of one or more of the following criteria.
  - Proteinuria with or without any of the following features [14]:
    - presence of haematuria and/or red cell casts,
    - increase in serum creatinine and/or decline in eGFR.
- Proteinuria was measured as a spot morning urine protein creatinine index (uPCI) and was positive if the value was >100 mg/mmol creatinine (NR ≤ 20).
  (II) Renal SLEDAI score ≥ 4 (out of 16) [15].
- Relapse/flare of LN was defined in two ways.
  - At recruitment, relapse was defined as recurrence of renal disease activity after a period of remission ≥3 months [14].
  - During this study period with only 4 months of observation (due to time constraints), relapse was defined as an increase in proteinuria and/or haematuria and/or serum creatinine level after 4 weeks of CR/PR or decrease in serum albumin level after 4 weeks of CR/PR [14].
- Remission was also defined in two ways.
  - At recruitment, remission was defined as absence or reduction of renal disease activity and no change in immunosuppressive therapy for at least 3 months [14].
  - In this study period with only 4 months of observation, remission was defined as absence or reduction of renal disease activity and no change in immunosuppressive therapy for at least 4 weeks [14].
- Inactive LN was defined by the presence of one or more of the following criteria.
  - Proteinuria (uPCI) ≤ 50 mg/mmol with/without any of the following features [14]:
    - serum albumin ≥ 35 g/L,
    - inactive urine sediments [no red blood cells (RBC < 5 red cells/HPF), no red cell casts and no leucocyturia (<5 white cells/HPF)],
    - stable serum creatinine (unless due to another aetiology, e.g., renin-angiotensin system (RAS) blockade).
  - Renal SLEDAI score 0 or < 4/16.

### 2.2. The Disease Course of LN

The disease course of LN was categorized at each visit using the definitions modified from Yamaji et al. [16] and Ruiz-Irastorza et al. [17] (Table 1).

1. Complete remission (CR).
2. Partial remission (PR).
3. Nonremission (NR) or unchanged.
4. Relapse/flare.

### 2.3. SLE Disease Activity Index and Laboratory Assessment

SLE Disease Activity Index (SLEDAI-2K) was used to assess lupus disease activity [15]. This index consists of three components: global (score range 0–150), renal (score range 0–16), and extrarenal (score range 0–63). The renal score corresponds to the presence of any one of the following on urinalysis: proteinuria, haematuria, leukocyturia, or urinary red cell casts after exclusion of stones or concurrent urinary tract infection or other causes of proteinuria [18].

Laboratory assessment included the following: full blood count, renal function test, estimated glomerular filtration rate (eGFR) using the Modification of Diet in Renal Disease (MDRD) formula, urinalysis, urine microscopy, urine protein creatinine index (uPCI), and serological tests (serum complement 3 and 4 levels (C3, C4) and anti-dsDNA antibody titres (anti-dsDNA Ab)).

### 2.4. Urine Sample Collection

A fresh urine sample (midstream) from each patient was collected in a sterile container. The urine was then transferred to 3 × 10 mL tubes. These were transported directly to the laboratory where they were centrifuged for 15 minutes at 1500 g to remove sediments then frozen in aliquots at −80°C for later uMCP-1 testing.

### 2.5. Method of Measuring Urinary MCP-1

CCL2/MCP-1 Quantikine ELISA KIT (R&D Systems USA) for urinary MCP-1 measurement was used. The Quantikine Human MCP-1 Immunoassay is a 3.5–4.5-hour solid phase ELISA designed to measure MCP-1 in cell culture supernates, serum, plasma, and urine. It contains* E. coli* expressed recombinant human MCP-1 and antibodies against the recombinant factor. It accurately quantifies recombinant human MCP-1. Results obtained show linear curves that are parallel to the standard curves obtained using the Quantikine kit standards.

## 3. Statistical Analysis

Categorical variables are presented as counts (percent). Continuous variables are presented as mean (±standard deviation (SD)) if normally distributed or median (interquartile range (IQR)) if nonnormally distributed. Pearson's chi-square test (*χ* ^2^) was used to compare categorical variables and a two-sided independent-sample *t*-test was used to compare normally distributed variables. Nonparametric tests (Mann–Whitney *U* and Kruskal–Wallis tests) were used for nonnormally distributed variables. Spearman's correlation coefficient was used to assess the association between uMCP-1 levels with standard laboratory parameters.

Receiver operating characteristic (ROC) curves were constructed to determine the performance characteristics of uMCP-1 levels for detection and prediction of LN activity. The best cutoff value for uMCP-1 was calculated based on maximization of the Youden index (sensitivity + specificity –1) [19]. Sensitivity, specificity, positive predictive value (PPV), and negative predictive value (NPV) of uMCP-1 as predictor of LN activity were also calculated.

Binary logistic regression analysis was performed to explore for independent predictors of LN activity. uMCP-1 and all standard markers of LN activity of the preceding visit with a *p* < 0.05 were included in the regression model. Data was analyzed using SPSS software version 18.0. Probability (*p*) values of <0.05 were considered significant.

## 4. Results

### 4.1. Characteristics of the Study Population

A total of 100 SLE patients with biopsy proven LN were recruited and all completed the 4-month observation period. The sociodemographic, clinical, and laboratory data between active and inactive LN groups are as shown in Table 2.

### 4.2. Course of LN in the Overall Study Population

At baseline, there were 47 patients with active LN (42 NR, 5 relapses) and 53 with inactive LN. The number with active LN decreased to 29 (27 NR, 2 relapses) at 2 months and to 22 (16 NR, 6 relapses) at 4 months, respectively, whereas the number of patients with inactive LN increased progressively from 53 at baseline to 71 (61 CR, 10 PR) at 2 months to 78 (59 CR, 19 PR) at 4 months, respectively. In summary, with time on treatment, the majority of patients with active LN achieved CR/PR although a few relapses occurred at each follow-up.

At each time point, there were significant differences between the active and inactive LN groups with regard to serum albumin (*p* < 0.01), proteinuria (uPCI, *p* < 0.001), SLEDAI-2K (global) (*p* < 0.001), and SLEDAI-2K (renal) (*p* < 0.001). At end study, serum creatinine had increased and eGFR declined significantly only in the active LN group. There were no differences between both groups in terms of anti-dsDNA Ab and serum complements (C3 and C4).

The detailed comparisons between active and inactive LN groups at each time point are summarized in Table 3. At all time points, uMCP-1 levels were significantly higher in the active group compared to those in the inactive LN group (Table 3).

#### 4.2.1. uMCP-1 Levels on Longitudinal Follow-Up

On longitudinal follow-up, uMCP-1 levels also differed significantly between those patients who attained CR/PR compared with those with NR or who relapsed.

At baseline, patients with CR/PR had median uMCP-1 levels of 3,682 pg/mg creatinine (IQR 6,426.05, range 0–23,866) compared with 7,499.33 pg/mg creatinine (IQR 11,303.36, range 548.30–40,170) in those with NR and 14,962.66 pg/mg creatinine (IQR 10,622, range 560–16,897) in those who relapsed (*p* = 0.002).

At 2 months, patients who achieved CR/PR had median uMCP-1 levels of 2,496 pg/mg creatinine (IQR 3,536.17, range 0–13,412) compared with 4,900 pg/mg creatinine (IQR 4,795 range 1,953.79–18,458) in those with NR and 9,654.66 pg/mg creatinine (range 8,711.33–10,538) in those who relapsed (*p* < 0.001).

At 4 months, patients who achieved CR/PR had median uMCP-1 levels of 2,220.84 pg/mg creatinine (IQR 2,028.17, range 0–11,470) compared with 7,288.50 pg/mg creatinine (IQR 5,507.61 range 1,208.69–13,716) in those with NR and 10,210.64 pg/mg creatinine (IQR 8,092, range 0–51,221) in those who relapsed (*p* < 0.001).

At each visit, uMCP-1 levels were highest in those patients with relapsed LN followed by the NR group and the lowest levels occurred in the remission group (CR/PR).

#### 4.2.2. Association between uMCP-1 with Parameters of LN Activity on Follow-Up

The associations of uMCP-1 with parameters of LN activity are summarized in Table 4.

### 4.3. Course of LN in the Group Active at Baseline

At baseline, the active LN group comprised 47 patients, 42 NR and five relapsers. At 2 months of follow-up, 18/47 (38%) achieved CR/PR, 27/47 (57.4%) had NR, and 2/47 (4%) relapsed. At 4 months, 28/47 (60%) achieved CR/PR, 16/47 (34%) had NR, and 3/47 (6%) relapsed (Table 5).

Two patients were subjected to repeat renal biopsy. In both, the histopathological findings had deteriorated from class II + V (Case 1) and class IV (Case 2) six months earlier to class III + V (both).

#### 4.3.1. uMCP-1 Levels and LN Activity on Follow-Up

The uMCP-1 levels decreased progressively from baseline to 2 months to end of study in response to treatment especially in those patients who achieved remission (Table 5). uMCP-1 levels were significantly lower in those who attained remission than in those with active LN (*p* < 0.001 in both).

### 4.4. Lupus Nephritis Relapses

**Figure 1:**
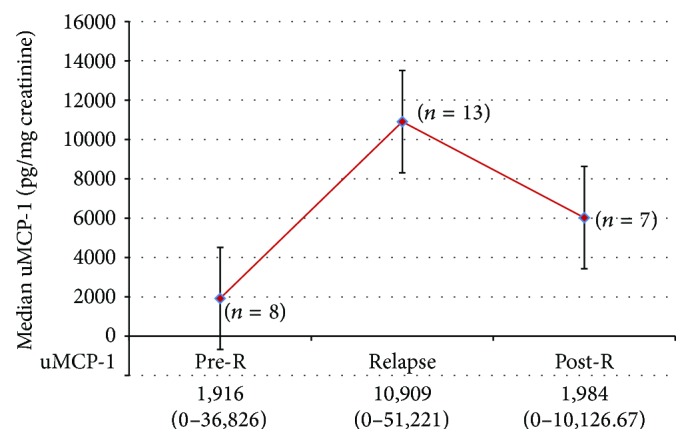
Median uMCP-1 levels in LN relapse compared to pre- and postrelapse levels.

On follow-up, 13 patients in the overall study population relapsed, five at baseline, two at 2 months, and six at 4 months, and were appropriately treated. Their median uMCP-1 levels were highest at the time of relapse compared to pre-relapse levels and decreased in response to treatment (Figure 1). Renal biopsy was repeated in 1/5 who relapsed at baseline and 1/2 at 2 months but in none of the six relapsers at 4 months. Their histological findings had deteriorated from class IV to class V and mixed class IV + V, respectively.

### 4.5. ROC Curve Analysis of uMCP-1 to Identify LN Activity

ROC curves were constructed to assess the potential diagnostic values of uMCP-1 compared with standard blood and urine markers at each visit to identify patients with active LN.

**Figure 2:**
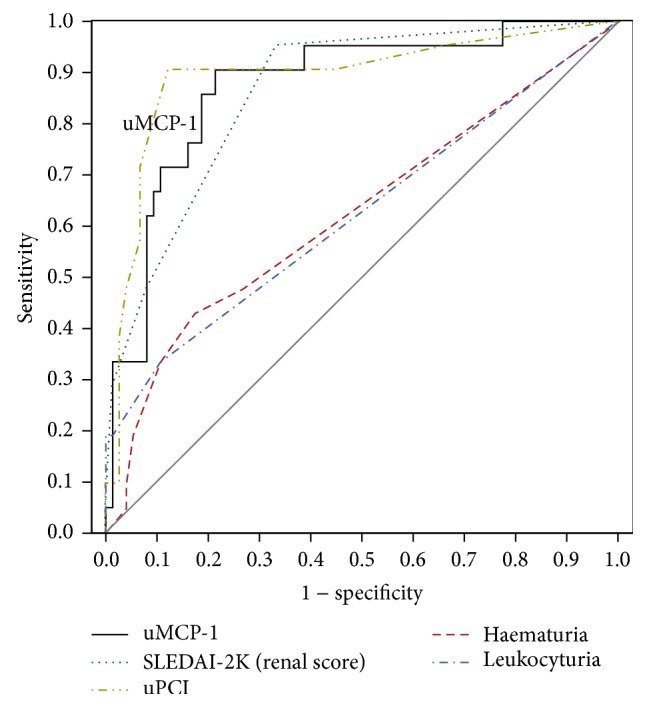
Receiver operating characteristic curve (ROC) of uMCP-1 compared with those of urinary parameters and SLEDAI-2K (renal) for the diagnosis of LN activity at 4 months. The black solid curve represents the uMCP-1; the area under the curve (AUC) was 0.87 (*p* < 0.001). The AUC for proteinuria was 0.89 (*p* < 0.001) and those for haematuria and leukocyturia were 0.62 (*p* = 0.07) and 0.62 (*p* = 0.08), respectively. The AUC for SLEDAI-2K was 0.85 (*p* < 0.001). Thus, uMCP-1 was better than haematuria and leukocyturia and essentially similar to proteinuria (uPCI) and SLEDAI-2K renal score for detection of LN activity at 4 months.

At each visit, the area under the curve (AUC) for uMCP-1 was higher than those for serum albumin, serum creatinine, eGFR, anti-dsDNA Ab titres, C3, and C4, for detection of LN activity (Table 6), whereas it was lower than those for proteinuria (uPCI) and SLEDAI-2K renal score. Thus, uMCP-1 was superior to most of the usual markers used for the monitoring of LN activity but was not as good as those for proteinuria (uPCI) and SLEDAI-2K renal score. This is illustrated by the ROC curves at end of study (Figure 2) which show that the AUC for uMCP-1 was very good at 0.87 (95% CI: 0.78–0.96: *p* < 0.001). At a maximum Youden index of 0.69, the cutoff value was 3,594 pg/mg creatinine. This gave a sensitivity of 0.90 and a specificity of 0.79, respectively.

### 4.6. Independent Predictors of LN Activity

Binary logistic regression was used to assess the independent predictors of LN activity. uMCP-1 and all relevant clinical variables with a *p* value ≤0.05 (Table 3) at 2 months were entered into the regression model to predict LN outcome at 4 months. These included serum albumin, serum creatinine, eGFR, proteinuria (uPCI), and SLEDAI-2K (renal) (Table 7). Only increasing proteinuria (uPCI) (OR = 4.93, 95% CI, 2.59–9.95, *p* = 0.03) and a fall in the serum albumin (OR = 0.83, 95% CI, 0.71–0.97, *p* = 0.02) emerged as independent predictors of LN activity.

A ROC curve was also constructed for uMCP-1 levels of the previous visit (i.e., at 2 months) to predict LN outcome at 4 months. The AUC for uMCP-1 was 0.77 (95% CI: 0.65–0.89; *p* < 0.001). At the maximum Youden index of 0.44, the cutoff value for uMCP-1 was 3,175 pg/mg creatinine. This gave a sensitivity of 0.86, specificity of 0.58, PPV of 0.37, and NPV of 0.94 for the prediction of LN activity.

## 5. Discussion

We have previously reported in a cross-sectional study that uMCP-1 levels were significantly elevated in patients with active LN compared to those with inactive renal disease [11]. On follow-up of our cohort, uMCP-1 levels were consistently higher in patients with active LN compared to those with inactive LN. The highest uMCP-1 levels were observed in those with renal relapses (*n* = 13) which their uMCP-1 levels decreased progressively with treatment. These findings are consistent with those reported to date from the few other longitudinal studies in the literature [8, 20, 21]. The Ohio SLE study followed 80 patients with SLE with and without LN and 28 healthy controls [8]. uMCP-1 levels were significantly higher in patients with renal flares (*n* = 25) than those with nonrenal flares (*n* = 22), SLE renal disease control subjects (*n* = 15), SLE nonrenal flare control subjects (*n* = 18), and healthy individuals (*n* = 28). uMCP-1 levels decreased over several months in patients who responded to treatment but were persistently high in nonresponders [8]. In another longitudinal study (*n* = 20), Singh et al. [20] reported that uMCP-1 could distinguish those patients with active LN from those with inactive renal disease or stable SLE. During follow-up, uMCP-1 levels decreased significantly in those patients who achieved remission (CR/PR) but did not change in nonresponders [20]. Torabinejad et al. [21] assessed the role of uMCP-1 and urinary transforming growth factor-*β*2 (uTGF-*β*2) in a longitudinal study involving 70 SLE patients and 10 healthy controls. They divided the SLE patients into 4 groups: 25 with active LN, 10 with remission LN, 25 with clinically active SLE and without LN, and 10 with SLE in remission and without LN. They demonstrated that the levels of both uMCP-1 and uTGF-*β*2 were significantly different in these groups. The highest levels were observed in the active LN group while the lowest were found in the controls. Both biomarkers decreased in response to treatment [21].

In our study patients, uMCP-1 levels correlated directly with proteinuria and inversely with serum albumin at recruitment and on follow-up. These findings corroborate with those reported in cross-sectional studies by Tucci et al. [22], Chan et al. [23], and Alzawawy et al. [24] and in a longitudinal study by Watson et al. [25]. Whereas Noris et al. [26] did not find this association.

At baseline and at 2 months, we found no association between uMCP-1 levels and serum creatinine or eGFR. Contradictory results have been reported in both cross-sectional studies [22, 23, 27, 28] and a longitudinal study by Rovin et al. [8]. However, at end study, uMCP-1 levels in our patients were found to be associated with serum creatinine and eGFR. Several reasons can account for this last observation: “mild” CKD progression, use of renin-angiotensin system (RAS) blockers in those patients with CR/PR, and relapse of LN (*n* = 13) which is often associated with an element of acute kidney injury (AKI).

We also found significant correlations between uMCP-1 with global SLEDAI-2K and renal SLEDAI-2K scores at all time points. Many authors had previously reported these findings in both cross-sectional studies [23, 27, 28] and in the longitudinal study by Rovin et al. [8].

At both follow-up visits, there were no associations between uMCP-1 levels and anti-dsDNA Ab titres. These findings concur with those reported by Watson et al. [25]. At 2 months, uMCP-1 levels were significantly associated with serum complements (C3, C4). The associations between uMCP-1 levels and serological markers remain controversial. El-Shehaby et al. [27] found uMCP-1 levels to be associated with serum complements C3 and C4 but not with anti-dsDNA Ab titres. Alzawawy et al. [24] (cross-sectional study, 30 SLE patients) and Kiani et al. [10] (longitudinal study, 87 SLE patients) reported that uMCP-1 levels and anti-dsDNA positivity were highly associated, whereas Watson et al. [25] (longitudinal study, 64 paediatric SLE patients) reported an association between uMCP-1 and serum C3.

At all time points, the ROC curves for uMCP-1 showed it to be a good consistent noninvasive marker for detection of LN activity. AUCs at all three visits were very good and ranged from 0.82 to 0.87 with sensitivities of 0.87–0.90 and specificities of 0.61–0.79. Torabinejad et al. [21] in their mixed SLE/LN cohort reported that uMCP-1 had an AUC of 0.90 with a sensitivity of 0.94 and specificity of 0.80 for diagnosis of LN regardless of SLE activity at baseline. In our study, uMCP-1 consistently outperformed the usual blood and urinary markers as well as the serological markers, that is, anti-dsDNA Ab titres and serum complements. However, uMCP-1 was not superior to proteinuria and SLEDAI-2K renal score for detection of LN activity. This may be due to the fact that both proteinuria and SLEDAI-2K renal score were included as major criteria in the definition of LN activity.

We also examined the ROC curve for uMCP-1 of the preceding visit which showed that a cutoff value of 3,175 pg/mg creatinine had a good sensitivity but lowish specificity for discriminating between active and inactive LN. Given the rather poor positive predictive value of 0.37, a uMCP-1 cutoff level of 3,175 pg/mg creatinine did not have the potential to predict LN activity. However, uMCP-1 levels of less than 3,175 pg/mg creatinine had the potential to predict the absence of LN activity with a negative predictive value of 94%.

In patients with LN active at baseline (*n* = 47), uMCP-1 levels fell significantly in response to treatment in all patients initially. In those who achieved CR/PR at end study (*n* = 28), uMCP-1 levels continued to decrease further, whereas, in those with persistent NR (*n* = 19), the uMCP-1 which fell initially rose again at end of study.

In the 13 patients with LN relapse, uMCP-1 levels not only increased concurrently with the relapse but also achieved the highest levels and then decreased progressively with treatment. In the one patient with NR at baseline who relapsed at 2 months with increasing proteinuria and rising serum creatinine levels despite increased treatment, her uMCP-1 levels rose in tandem. Interestingly, one patient who was initially in remission but relapsed at end study showed undetectable uMCP-1 levels throughout. This can perhaps be explained by MCP-1 gene polymorphism with her lacking the MCP-1 gene just like the MCP-1 knockout mice of the MRL/lpr lupus model [29] or her MCP-1 gene could have undergone mutation. Kim et al. [30] and Tucci et al. [22] had earlier reported MCP-1 gene polymorphism in SLE patients with LN except these authors had reported on the dominant allele and its predisposition to LN. Kim et al. [30] reported that a genetic polymorphism in the 5′ flanking region of the MCP-1 gene is associated with LN in SLE patients. Tucci et al. [22] reported that SLE patients with an A/G or G/G MCP-1–2518 genotype have a higher risk of developing LN.

Multiple logistic regression analysis showed that only proteinuria and serum albumin were independent predictors of LN activity or relapse but not uMCP-1. This may again be due to the fact that both proteinuria and serum albumin were included in the definition of LN activity. We hypothesize that had the definition also incorporated the histological class as well as the activity index (AI) and chronicity index (CI) of recent renal biopsies and these parameters entered into the regression model, uMCP-1 could well have emerged as an independent predictor. In this context, Chan et al. [23] found that uMCP-1 mRNA was significantly higher in patients with active LN than in those with inactive LN, or those with inactive nonrenal SLE and healthy controls. uMCP-1 mRNA correlated significantly with SLE disease activity indices and with the histological AI. However, uMCP-1 as measured by ELISA did not correlate with the histological AI.

Alternatively, the diagnostic performance of uMCP-1 could be improved when measured by the conventional assay method (ELISA) in combination with other urine proteins as demonstrated by Susianti et al. [31] or by using Multiplex bead assays (Luminex) which are able to detect a large panel of different cytokines in a single blood or urine sample [32]. In the literature, there are some data available on the use of multiplex bead assays for blood cytokine levels but very little data for urine cytokine levels [33]. Further studies are needed to validate this approach for the measurement of both blood and, particularly, urine cytokines [33]. Susianti et al. [31] assessed the role of urinary TGF-*β*1, MCP-1, NGAL, and IL-17 in adults with LN (*n* = 70). The patients were divided into 3 groups: 38 with severe LN (class III-IV LN patients), 12 with mild LN (class I-II LN patients), and 20 healthy controls. All biomarkers were measured by ELISA using a human kit for each biomarker. The authors found that all four biomarkers had good diagnostic performances. uNGAL had the best sensitivity and specificity followed by uMCP-1, uIL-17, and uTGF-*β*1. The best sensitivities and specificities were shown by the combination of uTGF-*β*1 and uNGAL followed by uMCP-1 and uNGAL.

As part of the overall thesis project, we have also compared uNGAL and uMCP1 in this patient cohort and found that both biomarkers showed good performances for detection of LN activity (data not shown and not previously published) [34]. However, the AUC values as well as sensitivities and specificities for uMCP-1 were greater than those for uNGAL. Thus, uMCP-1 appears superior to uNGAL as a noninvasive diagnostic marker for active LN. Nonetheless, these markers in combination may be superior to either use in isolation.

The performance of uMCP-1 can also conceivably be improved by using one of the system biology approaches “omics.” In general, these approaches are used for the universal detection of genes (genomics), mRNA (transcriptomics), proteins (proteomics), and metabolites (metabolomics) in a specific biological sample in a nontargeted and nonbiased manner [35]. He et al. [36] recently described the application of omics-based methodology for the study of kidney diseases. They discussed omics data integration in terms of improving early detection, predicting disease progression, and monitoring treatment response. Additionally, the omics tools may also improve our understanding of LN renal regulatory events and help identify new biomarkers and therapeutic targets [37].

In this modern era with the establishment of specialized SLE/LN centres, relapses and/or reactivation and/or NR have reduced in frequency and severity. Nonetheless, these remain a major issue in the management of LN patients. One reason for this is that the natural course of LN is typified by relapse-remission and to perform repeated renal biopsies for each LN relapse not only is highly traumatic but may lead to complications and is probably unethical beyond a certain maximal number in a given time frame. Thus, serial uMCP-1 monitoring in conjunction with the usual clinical parameters can obviate repeated “invasive” renal biopsies.

The other main reason for LN and/or reactivation and/or NR is that patient noncompliance which has only been recently recognized. Many studies have shown that significant nonadherence to medications occurs not only in renal transplant patients [38–40] but also in lupus patients leading to adverse outcomes [41–43]. In our cohort, several patients were nonadherent to the prescribed dose of corticosteroids or immunosuppressive drugs. In addition, they were also taking herbal and/or traditional medications. These included 3/13 of the relapsers and several with NR at recruitment. Despite repeated counseling on the importance of adherence to prescribed medications, one patient with NR at recruitment remained recalcitrant and suffered a relapse at end of study.

The main limitation of this study was the time lag between urine collections for uMCP-1 with initial renal biopsy. Thus, it was not possible to correlate uMCP-1 with the histological classes of LN. Another limitation was the (still) relatively small number of patients recruited and the short follow-up of only 4 months due to cost (predominantly) and time constraints.

In conclusion, uMCP-1 levels were markedly increased in those patients with active LN in particular those with renal relapse and correlated significantly with LN activity. uMCP-1 was able to distinguish active LN and/or relapse from inactive renal disease. It had consistently good diagnostic performances with a good sensitivity and moderate specificity for detection of LN activity and/or relapse. It also had a good sensitivity albeit lowish specificity for prediction of LN activity and/or relapse. Perhaps the usefulness of this biomarker could be improved by incorporating several other new markers currently also under study into a panel for assessing LN activity, somewhat similar to that recently validated by the FDA (USA) for acute kidney injury (AKI, NephroCheck). NephroCheck identifies the presence of 2 proteins (insulin-like growth-factor binding protein 7 (IGFBP7) and tissue inhibitor of metalloproteinases (TIMP-2)) in the urine of AKI patients.

Although uMCP-1 was not an independent predictor for LN activity, it could serve as an adjunctive marker if the clinical diagnosis of LN activity remains uncertain. Additionally, it may identify early relapse of LN, thus facilitating improved grading of LN activity in this complex disease leading to earlier treatment and better outcome. A larger, prospective, longitudinal study for a longer follow-up for at least of 2-3 years recruiting patients at the time of their renal biopsies is indicated.

## Figures and Tables

**Table 1:** Criteria for the definition of the course of lupus nephritis.

Outcome	Criteria			
	Proteinuria (uPCI)	Haematuria	Serum creatinine	Serum albumin
Complete remission (CR)	≤50 mg/mmol creatinine	<10 RBC × 10^6^/L + no RBC casts	Baseline or ≤25% increase	≥35 g/L
Partial remission (PR)	50% reduction in baseline or > 50 ≤ 300 mg/mmol creatinine	>10 < 50 RBC × 10^6^/L + no RBC casts	Baseline or ≤25% increase	≥35 g/L
Nonremission (NR)	No change or >300 mg/mmol creatinine	≥50 < 150 RBC × 10^6^/L ± RBC casts	≥25% increase	<35 g/L
Relapse/flare	Increase after 4 weeks of CR/PR	Increase after 4 weeks of CR/PR	Increase after 4 weeks of CR/PR	Decrease after 4 weeks of CR/PR

Adapted with modification from Yamaji et al. [16] and Ruiz-Irastorza et al. [17].

**Table 2:** Baseline demographic and characteristics in the active and inactive LN patient groups.

Parameters	All subjects *n* = 100	Active LN *n* = 47	Inactive LN *n* = 53	*p* value
Age, mean ± SD years	36.90 ± 10.62	36.40 ± 9.97	37.33 ± 11.24	0.74
Female: number (%)	92 (92%)	43 (91.5%)	49 (92.5%)	0.57
Male: number (%)	8 (8%)	4 (8.5%)	4 (7.5%)	
Race: number (%)				
Malay	41 (41%)	24 (51.1%)	17 (32.1%)	
Chinese	55 (55%)	21 (44.7%)	34 (64.2%)	0.14
Indian	4 (4%)	2 (4.3%)	2 (3.8%)	
LN duration in years	7 (1–24)	7 (1–24)	7 (1–17)	0.56
Mixed connective tissue disease (MCTD)	7 (7%)	3 (6.4%)	4 (7.5%)	0.82
Musculoskeletal system (MSK)	41 (41%)	20 (42.6%)	21 (39.6%)	0.46
Duration of MSK in years	6 (1–27)	6.5 (1–27)	6 (1–27)	0.60
Autoimmune Haemolytic Anaemia (AIHA)	26 (26%)	14 (29.8%)	12 (22.6%)	0.27
Duration of AIHA in years	4.88 ± 3.21	5.58 ± 3.44	4.28 ± 2.99	0.34
Idiopathic thrombocytopenic purpura (ITP)	9 (9%)	5 (10.6%)	4 (7.5%)	0.24
Duration of ITP in years	7.5 ± 4.62	9.5 ± 5.8	5.5 ± 2.38	0.20
Thrombotic thrombocytopenic purpura (TTP)	1 (1%)	0 (0%)	1 (1%)	0.53
Systolic blood pressure (mmHg)	128 ± 17.68	128 ± 13.16	120 ± 13.91	**0.001**
Diastolic blood pressure (mmHg)	75.2 ± 4.08	77.80 ± 10.31	73.68 ± 10.44	**0.04**
Classes of lupus nephritis (%)				
WHO class I	1 (1%)	1 (2.1%)	0 (0%)	
WHO class II ± V	6 (6%)	3 (6.4%)	3 (5.7%)	
WHO class III ± V	34 (34%)	15 (31.9%)	19 (35.8%)	0.71
WHO class IV ± V	52 (52%)	26 (55.3%)	26 (49.1%)	
WHO class V	5 (5%)	1 (2.1%)	4 (7.5%)	
WHO class VI	2 (2%)	1 (2.1%)	1 (1.9%)	
Activity index, median (IQR)	8 (0–19)	9 (0–16)	8 (0–19)	0.93
Chronicity index, median (IQR)	3 (0–15)	3.58 (0–9)	3 (1–15)	0.55
CKD stage (%)				
Stage 1 (eGFR > 90)	61 (61%)	25 (53.2%)	36 (67.9%)	
Stage 2 (eGFR 60–89)	22 (22%)	10 (21.3%)	12 (22.6%)	0.06
Stage 3 (eGFR 30–59)	14 (14%)	9 (19.1%)	5 (9.4%)	
Stage 4 (eGFR 15–29)	3 (3%)	3 (6.4%)	0 (0%)	
Medications, no (%)				
Corticosteroids	95 (95%)	43 (91.5%)	52 (98.1%)	0.12
Cumulative dose for previous six months (g)	1.80 (0.75–4.50)	1.80 (0.90–4.50)	1.76 (0.75–1.95)	**0.001**
Cumulative dose from previous relapse (g)	5.040 (0.90–24.43)	4.415 (0.90–24.43)	6.685 (1.59–13.32)	**0.009**
Time from last relapse (months)	22 (1–120)	11 (1–120)	28 (3.5–72)	**0.001**
Cyclophosphamide	8 (8%)	8 (17%)	0 (0%)	**0.002**
Cyclosporine A/Tacrolimus	30 (30%)	19 (40.4%)	11 (20.8%)	**0.03**
Mycophenolic acid	22 (22%)	12 (25.5%)	10 (18.9%)	0.42
Azathioprine	36 (36%)	12 (25.5%)	24 (45.3%)	**0.04**
Hydroxychloroquine	42 (42%)	20 (42.6%)	22 (41.5%)	0.91
Renin angiotensin system blockers (ACEI/ARB/spironolactone)	68 (68%)	29 (61.7%)	39 (73.6%)	0.11

SD: standard deviation; IQR: interquartile range; LN: lupus nephritis; WHO: World Health Organization; CKD: chronic kidney disease; ACEI: angiotensin converting enzyme inhibitors; ARBs: angiotensin receptor blockers; NS: not significant.

**Table 3:** Characteristics of patients with active and inactive LN at each time point.

Parameters	Baseline	2 months	4 months
Active LN (A)	A, *n* = 47	A, *n* = 29	A, *n* = 22
Inactive LN (IA)	IA, *n* = 53	IA, *n* = 71	IA, *n* = 78
Serum albumin	37.78 ± 5.54	39 ± 5	37.5 ± 4.97
(35–50 g/L)	41.88 ± 3.59	41.78 ± 3.20	41.02 ± 5.82
**Intergroup *p* value**	**<0.001**	**<0.001**	**0.01**

Serum creatinine	69 (IQR 33–252)	72 (IQR 30–244)	89.5 (IQR 43–244)
(44–80 *μ*mol/L)	63 (IQR 41–158)	65 (IQR 37–168)	63 (IQR 34–192)
**Intergroup *p* value**	0.29	0.36	**0.004**

eGFR	93.61 ± 46.01	91 ± 49.16	75.04 ± 39.95
(>60 mL/min/1.73 m^2^)	99.75 ± 31.54	98 ± 32.69	98.35 ± 35
**Intergroup *p* value**	0.43	0.53	**0.009**

ESR	38.5 (IQR 21–91)	41 (IQR 22–92)	32 (IQR 8–105)
(mm/hr)	33 (IQR 0–46)	49 (IQR 10–103)	36 (IQR 1–78)
**Intergroup *p* value**	0.37	0.86	0.36

Anti-dsDNA Ab titers	35.18 (IQR 1.73–195.97)	30.23 (IQR 0.74–267.61)	41.53 (IQR 2.07–291.62)
(<30 IU/dL)	24.24 (IQR 0.81–279.21)	14.37 (IQR 1.05–280)	18.90 (IQR 0.95–262.21)
**Intergroup *p* value**	0.84	0.89	0.73

Serum C3	100.5 ± 36.39	102.25 ± 40.53	94.26 ± 26.67
(79–152 mg/dL)	109.62 ± 39.94	106.37 ± 41.54	104.16 ± 33.04
**Intergroup *p* value**	0.24	0.32	0.21

Serum C4	21.46 ± 12.82	21.14 ± 10.95	22.81 ± 10.19
(16–38 mg/dL)	22.94 ± 11	23.95 ± 13.69	23.14 ± 9.48
**Intergroup *p* value**	0.54	0.32	0.89

Proteinuria (uPCI)	110 (IQR 10–510)	130 (IQR 10–480)	110 (IQR 10–510)
(<20 mg/mmol creatinine)	20 (IQR 10–50)	20 (IQR 10–50)	20 (IQR 10–30)
**Intergroup *p* value**	**<0.001**	**<0.001**	**<0.001**

Urinary leucocytes/HPF ×10^6^/L	0 (0–20)	0 (0–20)	0 (0–20)
	0 (0–5)	0 (0–15)	0 (0–10)
**Intergroup *p* value**	**<0.001**	0.30	**0.007**

Urinary	0 (0–10)	0 (0–20)	0 (0–50)
RBC/HPF ×10^6^/L	0 (0–5)	0 (0–15)	0 (0–30)
**Intergroup *p* value**	**<0.001**	0.40	**0.03**

uMCP-1	9,317 (IQR 548–40,170)	5,163 (IQR 1,953.79–18,458)	7,288 (IQR 0–51,221)
(pg/mg creatinine)	3,682 (IQR 0–23.866)	2,496 (IQR 0–13,412)	2,220 (IQR 0–11,470)
**Intergroup *p* value**	**<0.001**	**<0.001**	**<0.001**

SLEDAI-2K	8 (IQR 0–18)	6 (IQR 0–18)	8 (IQR 0–20)
(global: 0–105)	2 (IQR 0–10)	2 (IQR 0–12)	2 (IQR 0–17)
**Intergroup *p* value**	**<0.001**	**<0.001**	**<0.001**

SLEDAI-2K	4 (IQR 0–16)	4 (IQR 0–12)	4 (IQR 0–16)
(renal: 0–16)	0 (IQR 0–3)	0 (IQR 0–8)	0 (IQR 0–12)
**Intergroup *p* value**	**<0.001**	**<0.001**	**<0.001**

SLEDAI-2K	4 (IQR 0–12)	2 (IQR 0–10)	4 (IQR 0–12)
(extrarenal: 0–89)	2 (IQR 0–10)	2 (IQR 0–9)	2 (IQR 0–8)
**Intergroup *p* value**	0.66	0.18	0.10

**Table 4:** Association of uMCP-1 with parameters of LN activity on follow-up.

Spearman's rho variable (active : inactive LN)	Baseline (47 : 53)		2 months (29 : 71)		4 months (22 : 78)	
	*r*_sp_	*p* value	*r*_sp_	*p* value	*r*_sp_	*p* value
Serum albumin	−0.35	** 0.001**	−0.32	**0.001**	−0.22	**0.03**
Serum creatinine	0.09	0.38	0.14	0.15	0.24	**0.01**
eGFR	−0.10	0.30	−0.15	0.12	−0.24	**0.01**
Anti-dsDNA Ab titers (IU)	−0.04	0.64	−0.19	0.06	0.01	0.89
C3 (mg/dL)	−0.09	0.34	−0.29	** 0.003**	−0.04	0.70
C4 (mg/dL)	0.02	0.80	−0.23	** 0.02**	−0.01	0.86
Proteinuria (uPCI)	0.39	** 0.001**	0.48	**<0.001**	0.41	**<0.001**
Leukocyturia	0.26	** 0.008**	0.21	** 0.03**	0.19	0.06
Haematuria	0.13	0.18	0.09	0.38	0.11	0.24
SLEDAI-2K global score	0.27	** 0.006**	0.42	**<0.001**	0.29	**0.004**
SLEDAI-2K renal score	0.39	** 0.001**	0.43	**<0.001**	0.35	**0.001**
SLEDAI-2K-extrarenal score	−0.08	0.42	−0.18	0.74	−0.11	0.27

**Table 5:** Follow-up characteristics of the patient subgroup with LN active at baseline.

Parameters	Baseline	2 months	4 months
Active LN (A)	A, *n* = 47	A, *n* = 29	A, *n* = 19
Inactive LN (IA)		IA, *n* = 18	IA, *n* = 28
Systolic blood pressure	128 ± 13.16	131.69 ± 12.62	130.23 ± 12.51
(mmHg)		121.86 ± 11.73	123.33 ± 11.87
**Intergroup *p* value**		**0.01**	**0.01**

Diastolic blood pressure	77.80 ± 10.31	80.73 ± 10.76	81.15 ± 7.86
(mmHg)		72.73 ± 8.25	73.14 ± 9.01
**Intergroup *p* value**		**0.03**	0.77

Serum albumin	37.78 ± 5.54	37.60 ± 5.02	36.92 ± 2.53
(35–50 g/L)		40.93 ± 2.54	40.66 ± 3.74
**Intergroup *p* value**		**0.01**	**0.01**

Serum creatinine	69 (IQR 33–252)	81.97 (IQR 40–244)	86 (IQR 48–224)
(44–80 *μ*mol/L)		67 (IQR 44–139)	62 (IQR 41–143)
**Intergroup *p* value**		0.43	**0.01**

eGFR	93.61 ± 46.01	88.56 ± 40.88	71.15 ± 29.20
(60 mL/min/1.73 m^2^)		97.93 ± 31.81	99 ± 38.15
**Intergroup *p* value**		0.28	**0.01**

ESR	38.5 (IQR 21–91)	45 (IQR 22–92)	32 (IQR 8–105)
(mm/hr)		55 (IQR 10–103)	36.50 (IQR 1–70)
**Intergroup *p* value**		0.48	0.78

Anti-dsDNA Ab titers	35.18 (IQR 1.73–195.97)	38.59 (IQR 0.74–267.61)	13.75 (IQR 2.11–175.22)
(<30 IU)		13.82 (IQR 1.54–135.29)	41.53 (IQR 2.07–252.85)
**Intergroup *p* value**		0.94	0.82

Serum C3	100.5 ± 36.39	96.44 ± 32.54	106.06 ± 39.29
(79–152 mg/dL)		113 ± 43.05	98.25 ± 21.99
**Intergroup *p* value**		0.43	0.50

Serum C4	21.46 ± 12.82	20.08 ± 10.12	22.15 ± 19.90
(16–38 mg/dL)		28.52 ± 16	22.93 ± 11.77
**Intergroup *p* value**		0.20	0.31

Proteinuria (uPCI)	110 (IQR 10–510)	120 (IQR 10–480)	110 (IQR 10–510)
(<20 mg/mmol creatinine)		30 (IQR 10–50)	40 (IQR 10–50)
**Intergroup *p* value**		**<0.001**	**<0.001**

Urinary leucocytes/HPF ×10^6^/L	0 (IQR 0–20)	0 (IQR 0–20)	0 (IQR 0–20)
		0 (IQR 0–10)	0 (IQR 0–5)
**Intergroup *p* value**		0.31	**0.009**

Urinary RBC/HPF ×10^6^/L	0 (IQR 0–10)	0 (IQR 0–20)	0 (IQR 0–50)
		0 (IQR 0–5)	0 (IQR 0–20)
**Intergroup *p* value**		0.29	0.23

uMCP-1	9,317 (IQR 548–40,170)	5,031 (IQR 1,953.79–18,408)	7,092.95 (IQR 1,208.69–17,070)
(pg/mg creatinine)		2,955 (IQR 0–12,920)	2,202.16 (IQR 0–10,573)
**Intergroup *p* value**		**<0.001**	**0.001**

SLEDAI-2K	8 (IQR 0–18)	6 (IQR 0–18)	8 (IQR 4–16)
(global: 0–105)		0 (IQR 0–12)	2 (IQR 0–12)
**Intergroup *p* value**		**<0.001**	**<0.001**

SLEDAI-2K	4 (IQR 0–16)	4 (IQR 0–12)	4 (IQR 4–16)
(renal: 0–16)		0 (IQR 0–8)	0 (IQR 0–12)
**Intergroup *p* value**		**<0.001**	**<0.001**

SLEDAI-2K	4 (IQR 0–12)	2 (IQR 0–10)	4 (IQR 0–8)
(extrarenal: 0–89)		0 (IQR 0–8)	2 (IQR 0–4)
**Intergroup *p* value**		0.10	0.65

**Table 6:** Area under the curve (AUC) of ROC curves for uMCP-1 and standard biomarkers for LN activity on longitudinal follow-up.

Variables	Baseline (95% CI)				2 months (95% CI)				4 months (95% CI)			
	AUC	*p*	LB	UB	AUC	*p*	LB	UB	AUC	*p*	LB	UB
uMCP-1	0.82	**0.001**	0.73	0.91	0.82	**<0.001**	0.73	0.90	0.87	**<0.001**	0.78	0.95
Serum albumin	0.25	**0.001**	0.13	0.35	0.23	** <0.001**	0.13	0.33	0.21	** <0.001**	0.10	0.31
Serum creatinine	0.58	0.21	0.44	0.71	0.55	0.42	0.41	0.69	0.68	**0.009**	0.54	0.82
eGFR	0.41	0.21	0.28	0.55	0.42	0.22	0.28	0.55	0.31	**0.01**	0.17	0.45
Anti-dsDNA Ab titres	0.50	0.96	0.37	0.63	0.49	0.78	0.35	0.62	0.56	0.46	0.38	0.73
Serum C3	0.37	0.50	0.25	0.50	0.45	0.84	0.33	0.57	0.40	0.21	0.25	0.54
Serum C4	0.43	0.26	0.30	0.56	0.42	0.22	0.29	0.54	0.45	0.53	0.29	0.61
Proteinuria (uPCI)	0.94	**<0.001**	0.89	0.98	0.92	**<0.001**	0.86	0.99	0.89	**<0.001**	0.80	0.98
Haematuria	0.72	**0.001**	0.60	0.84	0.54	0.49	0.41	0.67	0.62	0.07	0.48	0.77
Leukocyturia	0.65	0.23	0.52	0.77	0.54	0.51	0.41	0.67	0.62	0.08	0.47	0.77
SLEDAI-2K (renal score)	0.96	**<0.001**	0.71	0.90	0.84	**<0.001**	0.76	0.93	0.85	**<0.001**	0.77	0.94

**Table 7:** Predictors of LN outcome at 4 months' follow-up.

Variables	*β*	S.E	*p*	OR	95% CI for Exp(*β*)	
					Lower	Upper
uMCP-1	0	0	0.15	1.000	1.000	1.000
Serum albumin	−0.18	0.08	**0.02**	0.83	0.71	0.97
Serum creatinine	0.001	0.01	0.97	1.001	0.96	1.03
eGFR	−0.005	0.01	0.70	0.99	0.96	1.02
Proteinuria (uPCI)	11.98	5.63	**0.03**	4.93	2.59	9.95
SLEDAI-2K (renal score)	0.09	0.11	0.45	1.09	0.86	1.37

*R* ^2^ 0.39 (Hosmer and Lemeshow's), 0.32 (Cox and Snell), and 0.49 (Nagelkerke). Model *x* ^2^ = 38.14, *p* < 0.001. *β*: beta; SE: standard error; OR; odds ratio = Exp(β); CI: confidence interval.
